# Serotonin modulates glutamatergic transmission to neurons in the lateral habenula

**DOI:** 10.1038/srep23798

**Published:** 2016-04-01

**Authors:** Guiqin Xie, Wanhong Zuo, Liangzhi Wu, Wenting Li, Wei Wu, Alex Bekker, Jiang-Hong Ye

**Affiliations:** 1 Department of Anesthesiology, Pharmacology and Physiology, Rutgers, The State University of New Jersey, New Jersey Medical School, Newark, New Jersey, USA

## Abstract

The lateral habenula (LHb) is bilaterally connected with serotoninergic raphe nuclei, and expresses high density of serotonin receptors. However, actions of serotonin on the excitatory synaptic transmission to LHb neurons have not been thoroughly investigated. The LHb contains two anatomically and functionally distinct regions: lateral (LHbl) and medial (LHbm) divisions. We compared serotonin’s effects on glutamatergic transmission across the LHb in rat brains. Serotonin bi-directionally and differentially modulated glutamatergic transmission. Serotonin inhibited glutamatergic transmission in higher percentage of LHbl neurons but potentiated in higher percentage of LHbm neurons. Magnitude of potentiation was greater in LHbm than in LHbl. Type 2 and 3 serotonin receptor antagonists attenuated serotonin’s potentiation. The serotonin reuptake blocker, and the type 2 and 3 receptor agonists facilitated glutamatergic transmission in both LHbl and LHbm neurons. Thus, serotonin via activating its type 2, 3 receptors, increased glutamate release at nerve terminals in some LHb neurons. Our data demonstrated that serotonin affects both LHbm and LHbl. Serotonin might play an important role in processing information between the LHb and its downstream-targeted structures during decision-making. It may also contribute to a homeostatic balance underlying the neural circuitry between the LHb and raphe nuclei.

The brainstem dorsal and medial raphe nuclei, cell groups B7 and B8, respectively^1^, are the main source of forebrain serotonergic innervation^2^,^3^,^4^. Serotonin (5-HT) exerts differential effects by a variety of receptor subtypes (5-HT_1–7_). It is implicated in a broad spectrum of functions, including the regulation of appetite, locomotion, stress response, emotional and social behavior, cognition and associative learning, the sleep-wake cycle, reward-related behaviors, and the etiology of psychiatric disorders, such as schizophrenia and major depression^5^,^6^.

The lateral habenula (LHb) has emerged as a key brain structure in the pathophysiology of depression^7^,^8^,^9^,^10^ and decision making^11^. The LHb is composed of lateral (LHbl) and medial (LHbm) divisions that are anatomically and functionally heterogeneous with different connectivity. The LHbl receives inputs mainly from the basal ganglia^12^ and sends outputs through the rostromedial tegmental nucleus (RMTg) mainly to the dopaminergic neurons in the ventral tegmental area and substantia nigra and the serotoninergic neurons in the raphe nuclei^13^,^14^. The LHbm receives inputs from the limbic areas and sends outputs mainly to the GABAergic interneurons in the raphe nuclei^15^,^16^. Hence, changes in the activity of LHb neurons in these subregions may lead to different reactions in their targeted brain areas.

Anatomical evidence has revealed a strong reciprocal innervation between the LHb and the raphe nuclei^17^,^18^,^19^,^20^, and a high density of 5-HT receptors are expressed in the LHb^21^,^22^,^23^,^24^,^25^,^26^ suggesting a candidate regulatory pathway consists of serotonergic afferents from the raphe nuclei to the LHb. Indeed, there is functional evidence that 5-HT^12^, as well as activation of 5-HT_2C_^27^ and 5-HT_1B_^28^ receptors affect glutamate transmission and/or activity of LHb neurons. Moreover, we recently demonstrated that 5-HT induces an excitatory inward current in the LHb. Interestingly, whereas this inward current in LHbl neurons is larger than that in LHbm neurons, 5-HT-induced increase in firing rate is similar in these two subregions^29^, suggesting that the postsynaptic excitatory effect of 5-HT may be counteracted by its effects on synaptic transmissions. However, actions of 5-HT on the excitatory synaptic transmission to LHb neurons have not been thoroughly investigated.

In this study, we examined the effects of 5-HT on the glutamate transmission in the LHb, and whether differences in the effects of 5-HT existed between the LHbm and LHbl neurons. We also examined the receptor subtypes that mediate 5-HT-induced facilitation of glutamate transmission.

## Results

### 5-HT decreases glutamate transmission in a subset of LHbm and LHbl neurons

**Figure 1:**
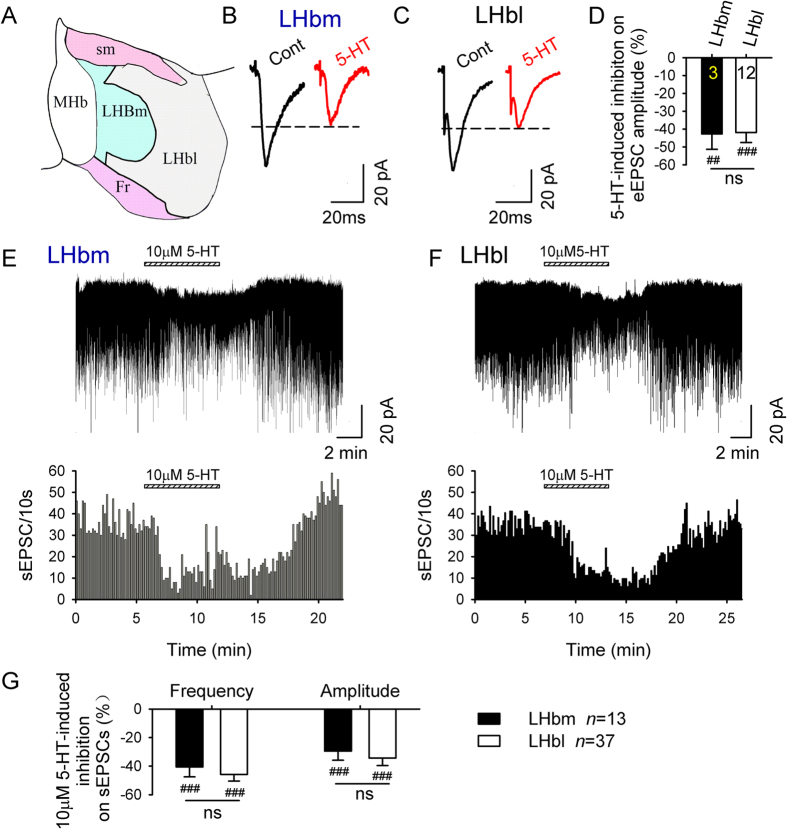
5-HT reduces glutamate transmission in some LHbm and LHbl neurons. (**A**) A scheme of the delineation of individual LHb subnuclei (modified from Fig. 3E^30^). Bath application of 10 μM 5-HT substantially suppressed EPSCs evoked by electrical stimulation in an LHbm (**B**) and an LHbl (**C**) neurons. Data are averages of 10 traces. (**D**) Summary of 10 μM 5-HT-induced inhibition on eEPSC amplitude in LHbm and LHbl neurons. 10 μM 5-HT greatly reduced sEPSCs in an LHbm (**E**) and an LHbl (**F**) cells. Horizontal bar above the current trace signals the application of the indicated drug. (**G**) Summary of 5-HT-induced % inhibitions on sEPSC frequency and amplitude. ^##^*p* < 0.01, ^###^*p* < 0.001, Student’s paired *t*-test for 5-HT vs baseline. ns means no significant difference between LHbm and LHbl neurons, unpaired *t*-test. Numbers of cells are indicated.

We identified the LHbm and LHbl according to previous reports^30^,^31^ (Fig. 1A). To compare the effects of 5-HT on glutamate transmission in LHbm and LHbl neurons, we first examined the effect of 5-HT on eEPSCs evoked by a local electrode. 5-HT (10 μM) markedly suppressed eEPSC amplitude in some neurons of the two subregions (Table 1, Fig. 1B–D). We then examined spontaneous EPSCs (sEPSCs). Neurons in the LHbl and LHbm had similar average basal sEPSC frequencies (LHbm, 1.9 ± 0.2 Hz, *n* = 141; LHbl, 2.1 ± 0.3 Hz, *n* = 97; LHbm vs LHbl: *t* = 0.78, *p* = 0.43) and amplitudes (LHbm, 16.8 ± 1.1 pA, *n* = 141; LHbl, 18.1 ± 1.2 pA, *n* = 97; LHbm vs LHbl: *t* = 0.8, *p* = 0.45). 5-HT inhibited sEPSCs in some neurons in these two subregions (Table 1, Fig. 1E–G).

### 5-HT facilitates glutamate transmission in many LHb neurons

**Figure 2:**
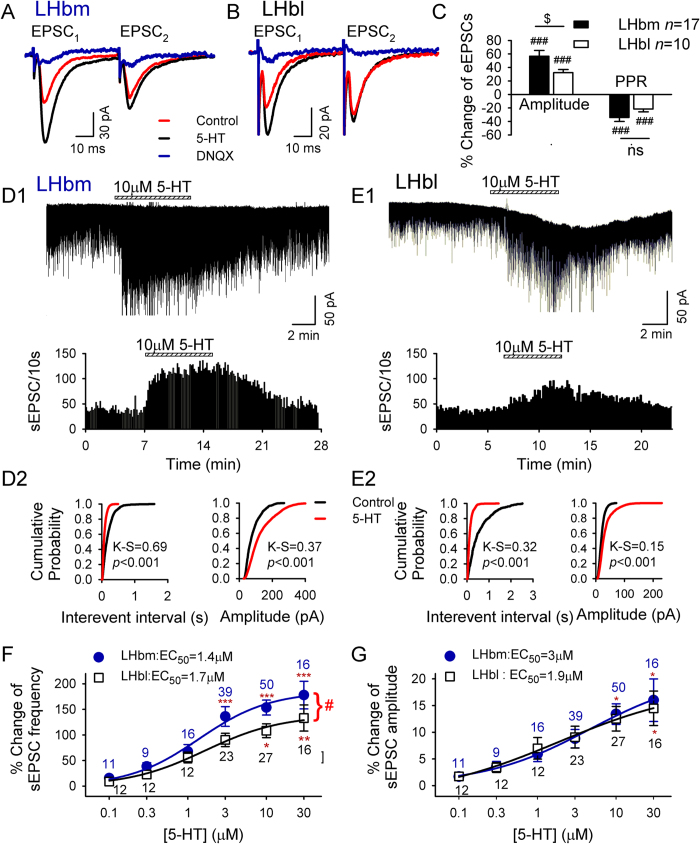
5-HT-induced potentiation of glutamate transmission is stronger in LHbm neurons than in LHbl neurons. Two EPSCs were evoked by twin-pulses (50 msec apart) stimuli, and 5-HT increased the first (EPSC_1_) but not second (EPSC_2_) of each pair in LHbm (**A**) and LHbl (**B**) cells. 20 μM DNQX abolished the current. (**C**) Percent change in amplitude and paired-pulse ratio (PPR = EPSC_2_/EPSC_1_) of eEPSCs. ^###^*p* < 0.001, Student’s paired *t*-test for 5-HT vs baseline. ^$^*p* < 0.05, unpaired *t*-test. (**D1–E1)** Upper: A representative example of increased sEPSCs by 10 μM 5-HT in an LHbm (**D1**) or an LHbl (**E1**) neuron. Lower: Time course of 5-HT’s effect on sEPSC frequency. Cumulative probability plots show higher incidence of shorter inter-sEPSC intervals and larger sEPSCs during 5-HT applications (red line) in the LHbm (**D2**) and LHbl (**E2**) neurons. Concentration-dependent increases in sEPSC frequency (**F**) and amplitude (**G**) in LHbm (•) and LHbl (▫) neurons. The smooth curve is the best fit to the data by the logistic equation. *p < 0.05, **p < 0.01, ***p < 0.001, two-way ANOVA followed by Tukey’s multiple comparisons test. ^#^*p* < 0.05 between LHbm and LHbl.

Interestingly, in contrast to the inhibition of EPSCs described above and by others^12^,^28^, we found 5-HT increased glutamate release in many LHb neurons, and the potentiation was greater in LHbm than LHbl neurons. Specifically, 10 μM 5-HT induced a significantly greater potentiation in eEPSC amplitude in 17/21 LHbm neurons than in 10/24 LHbl neurons (LHbm vs LHbl: *t* = 2.1, *p* = 0.048; Fig. 2A–C). In a similar manner, 10 μM 5-HT induced a significantly greater increase in sEPSC frequency in 50/67 LHbm neurons than in 27/70 LHbl neurons (LHbm vs LHbl: *t* = 2, *p* = 0.046; Fig. 2D–F, Table 1). In the remaining LHb neurons, 5-HT did not induce an observable response on eEPSCs (LHbm: *n* = 1/21; LHbl: *n* = 2/24; Table 1) or sEPSCs (LHbm: *n* = 4/67; LHbl: *n* = 6/70; Table 1).

Moreover, 5-HT potentiated EPSCs in the majority of the LHbm neurons (eEPSCs: *n* = 17/21; sEPSCs: *n* = 50/67), but in less than half of the LHbl neurons (eEPSCs: *n* = 10/24; sEPSCs: *n* = 27/70). The difference was significant (eEPSCs: Chi-square = 7.4 with df 2, *p* = 0.025; sEPSCs: Chi-square = 18.7 with df 2, *p* < 0.001). The deferential modulation of glutamate transmission by 5-HT (10 μΜ) in the LHbm and LHbl is summarized in Table 1. Notably, 5-HT inhibited EPSCs in significantly higher percentage of LHbl neurons than LHbm neurons (*p* < 0.001). By contrast, 5-HT potentiated EPSCs in significantly higher percentage of LHbm neurons than LHbl neurons (*p* < 0.001). Since our data of 5-HT inhibition of EPSCs generally agree with previous reports^12^,^28^, and there is no previous report on 5-HT potentiation of EPSCs, the present study was designed to investigate the mechanisms involved in the 5-HT-induced potentiation of EPSCs.

### 5-HT’s enhancement of sEPSCs is greater in LHbm than in LHbl neurons

5-HT significantly increased the frequency and amplitude of sEPSCs in the LHbm and LHbl neurons (Fig. 2D1,E1). This effect, also indicated by the increased incidence of shorter inter-sEPSC intervals (Fig. 2D2,E2 left panels), was reversible by washout; and was significantly stronger in LHbm neurons than in LHbl neurons (main effect of subregions, *F*_1, 231_ = 5.2, *p* = 0.024; Fig. 2F), as revealed by Two way ANOVA. 5-HT’s action was concentration dependent (main effect of doses, *F*_5, 231_ = 13.5, *p* < 0.001), with the EC_50_s of 1.4 ± 0.4 μM for LHbm neurons and 1.7 ± 0.4 μM for LHbl neurons. *Post-hoc* tests showed that sEPSC frequencies were significantly increased from baseline following a moderate dose of 5-HT administration (LHbm ≥ 3 μM, LHbl ≥ 10 μM). The increase of sEPSC frequency induced by 3 μM (*p* = 0.042) or 10 μM (*p* = 0.029) 5-HT were significantly greater in LHbm neurons than in LHbl neurons (Fig. 2F). However, there was no significant difference on the subregions × doses interaction (*F*_5, 231_ = 0.4, *p* = 0.85).

5-HT-induced increase in sEPSC frequency was accompanied by a higher incidence of larger sEPSCs (K-S test, Fig. 2D2,E2 right panels) in a concentration-dependent manner, with EC_50_s of 3 ± 1.5 μM in LHbm cells and 1.9 ± 0.9 μM in LHbl cells (*F*_5,231_ = 7.4, *p* < 0.001;Two-way ANOVA; Fig. 2G). No significant difference was detected between these subregions (no effect of subregions, *F*_1, 231_ = 0.02, *p* = 0.9, or subregions × doses interaction, *F*_5, 231_ = 0.07, *p* = 0.99).

### 5-HT facilitates glutamate transmission via a presynaptic mechanism

**Figure 3:**
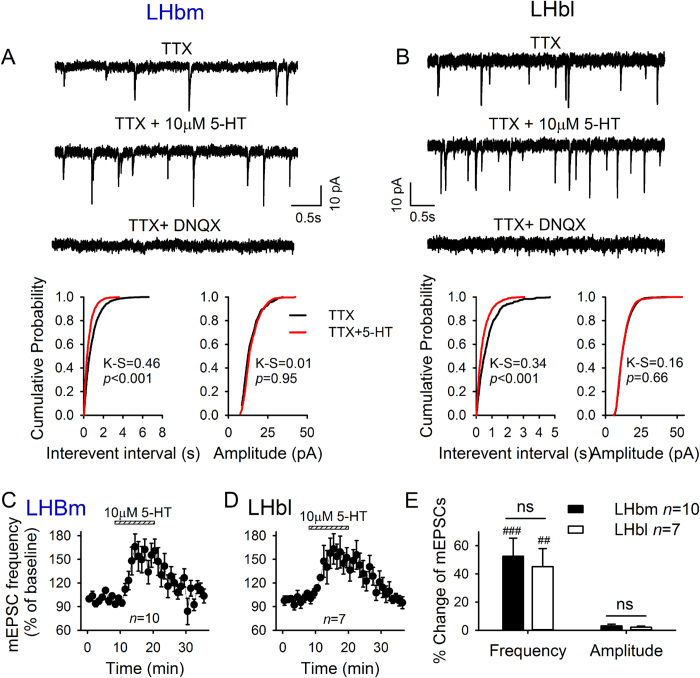
5-HT enhances glutamate transmission on LHb neurons by presynaptic mechanisms. Exemplar current traces showing facilitation of mEPSCs by10 μM 5-HT in a LHbm (**A**) and a LHbl (**B**) neuron in the presence of 0.5 μM TTX, 10 μM gabazine, and 1 μM strychnine. 20 μM DNQX completely abolished these mEPSCs. Cumulative probability plots show 5-HT increased incidence of shorter interval, but did not alter the amplitude of mEPSCs from the same neuron. Time course of 5-HT-induced changes in mEPSC frequency in the LHbm (**C**) and the LHbl (**D**) subnuclei. (**E**) Summary of 5-HT-induced changes (%) on mEPSCs.

To investigate the mechanisms of 5-HT-induced potentiation of EPSCs, we measured the amplitude of EPSCs evoked by twin pulses of 50 milliseconds apart by a local electrode and the paired pulse ratio (PPR = EPSC_2_/EPSC_1_). 5-HT (10 μM) robustly enhanced the amplitude of the first EPSC (EPSC_1_) of each pair (Fig. 2A,B), but not the second EPSC (EPSC_2_). Thus, 5-HT significantly reduced the paired pulse ratio (LHbm: 34.1 ± 5.9% decrease relative to baseline, *n* = 17, *p* < 0.001; LHbl: 21.6 ± 4.1%, *n* = 10, *p* < 0.001; LHbm vs LHbl: *t* = 1.5, *p* = 0.15; Fig. 2C). These data suggested that 5-HT enhanced presynaptic glutamate release. This idea was further supported by the data of miniature EPSCs (mEPSCs) recorded in the presence of tetrodotoxin. 5-HT (10 μM) shifted the cumulative interevent interval distribution towards shorter intervals (*p* < 0.05, K-S test; Fig. 3A,B), thus significantly increasing the mean frequency (LHbm: *p* < 0.001, *n* = 10/12; LHbl: *p* < 0.01, *n* = 7/13; LHbm vs LHbl: *p* > 0.5, unpaired *t*-test; Fig. 3E). This effect was reversible by washout (Fig. 3C,D). 5-HT had no significant effect on mEPSC amplitude distribution (*p* > 0.05, K-S test; Fig. 3A,B) nor on mean mEPSC amplitude (both *p* > 0.05 vs baseline, paired *t*-test; LHbm vs LHbl: *p* > 0.5; Fig. 3E).

### 5-HT_2_ and 5-HT_3_ receptors mediate 5-HT’s potentiation of glutamate transmission in LHb neurons

**Figure 4:**
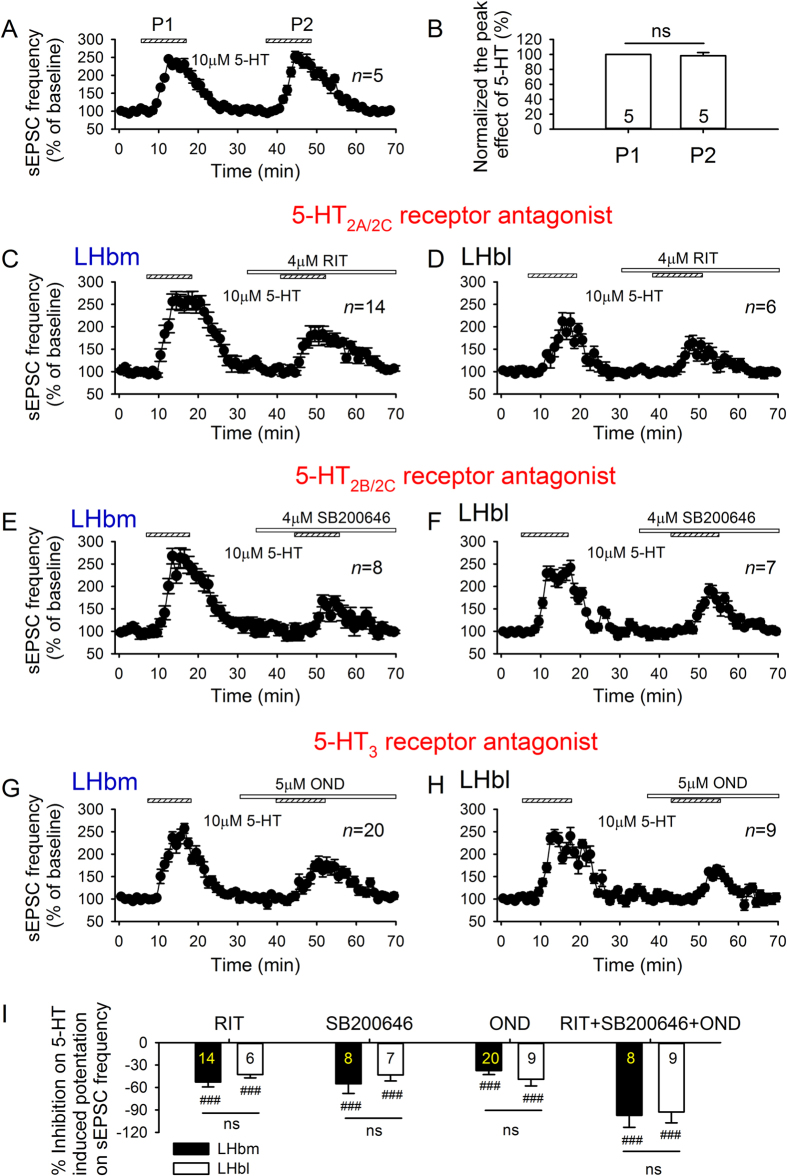
5-HT_2_ and 5-HT_3_ receptor mediate 5-HT-induced increase in sEPSC frequency. (**A,B**) Repeated applications of 5-HT (10 μM) induced similar facilitation of sEPSC frequency. Time courses of 5-HT-induced acceleration of sEPSCs in the absence and presence of 5HT_2A/2C_R antagonist RIT (**C**,**D**), 5-HT_2B/2C_R antagonist SB200646 (**E**,**F**), and 5-HT_3_R antagonist OND (**G**,**H**) in LHbm (**C**,**E**,**G**) and LHbl (**D**,**F**,**H**) neurons. (**I**) Summary of inhibition (%) by RIT, SB200646, OND or the cocktail containing RIT, SB200646 plus OND on the increase of sEPSC frequency induced by10 μM 5-HT in LHbm and LHbl cells. ^###^*p* < 0.001 vs 5-HT alone, Student’s paired *t*-test.

**Figure 5:**
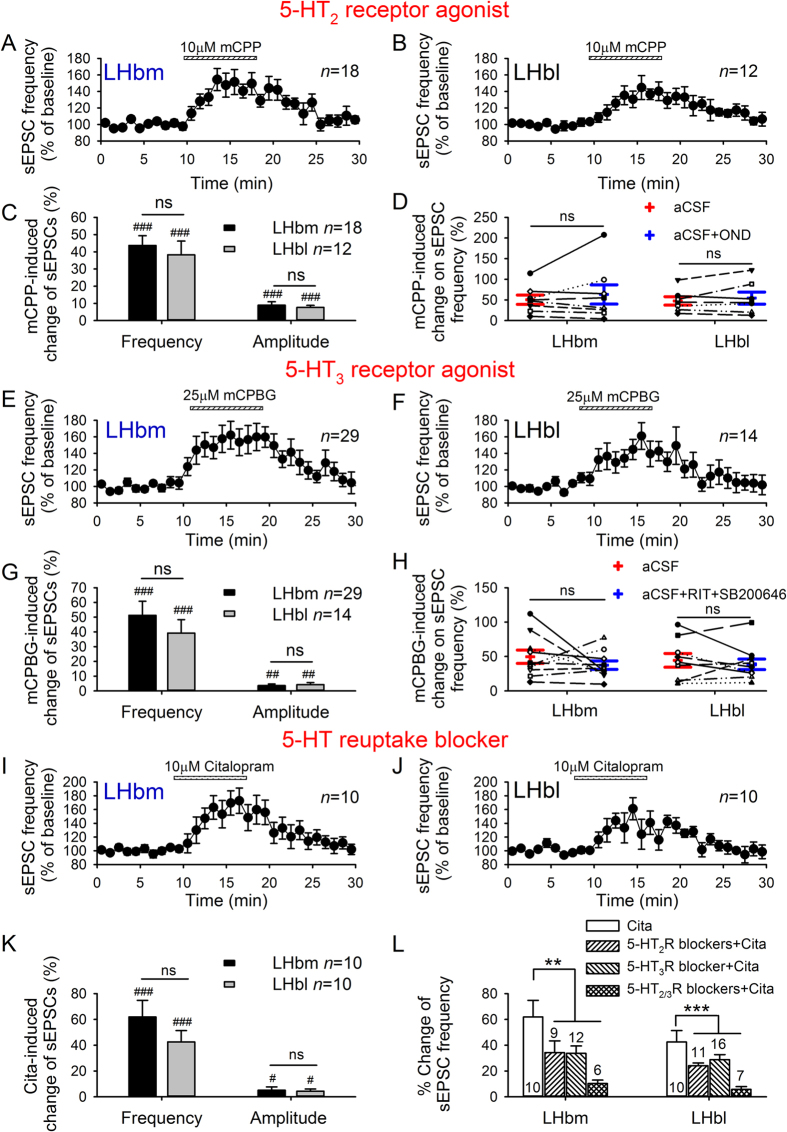
5-HT_2_ and 5-HT_3_ receptor agonists, and 5-HT reuptake blocker potentiate glutamate transmission in the LHb. Time course of facilitation of sEPSC frequency induced by 5-HT_2_R agonist (10 μM mCPP) in LHbm (**A**) and LHbl (**B**) neurons, and by 5-HT_3_R agonist (25 μM mCPBG) in LHbm (**E**) and LHbl (**F**) neurons. Mean (± SEM) of mCPP (**C**) or mCPBG (**G**)-induced increase on sEPSC frequency and amplitude. Mean ± SEM and individual values before and after 5-HT antagonists demonstrated that 5-HT_3_R antagonist (OND) or 5-HT_2_R antagonists (RIT plus SB200646) did not alter facilitation of sEPSC frequency induced by mCPP (**D**) or mCPBG (**H**). Time course of facilitation of sEPSC frequency induced by the 5-HT transport inhibitor citalopram (10 μM) in LHbm (**I**) and LHbl (**J**) neurons. (**K**) Mean (±SEM) of citalopram-induced increase on sEPSC frequency and amplitude. ^#^*p* < 0.05, ^##^*p* < 0.01, ^###^*p* < 0.001 vs baseline, Student’s paired *t*-test. ns, no significant difference between LHbm and LHbl neurons, unpaired *t*-test. (**L**) Mean (±SEM) potentiation of sEPSC frequency induced by citalopram in the absence and presence of 5-HT_2_ receptor antagonists (RIT plus SB200646), 5-HT_3_ receptor antagonist (OND) or the cocktail of 5-HT_2/3_ receptor antagonists. **p < 0.01, ***p < 0.001, One-way ANOVA followed by Tukey’s multiple comparisons test.

5-HT_2_ and 5-HT_3_ receptors mediate 5-HT’s excitatory effects in many brain areas, and histological and molecular evidence indicates that 5-HT_2_^26^ and 5-HT_3_ receptors^24^,^25^ exist in the LHb. The second application of 5-HT to the same neuron was equally effective in facilitating sEPSC frequency (Fig. 4A,B). Thus, 5-HT produced a reliable and pronounced increase in sEPSC frequency in the majority of LHb neurons. We compared the effects of 5-HT on sEPSC frequency in the absence and presence of antagonists of 5-HT_2_ and 5-HT_3_ receptors. After recovery from the facilitation of sEPSCs induced by the first 5-HT application, an antagonist of 5-HT_2A/C_ receptor (10 μM ritanserin (RIT)), 5-HT_2B/C_ receptor (4 μM SB200646), 5-HT_3_ receptor (5 μM ondansetron (OND)), or a combination of these (RIT + SB200646 + OND) were bath applied for 6–8 min before the second 5-HT application. These antagonists, when applied alone, did not significantly alter the basal sEPSCs (data not shown), but their presence substantially attenuated 5-HT-induced facilitation of sEPSCs (all *p* < 0.001 vs 5-HT alone, paired *t*-test; Fig. 4C–H). The attenuation in LHbm was similar to that in LHbl (all *p* > 0.25, unpaired *t*-test; Fig. 4I). Notably, 5-HT-induced facilitation was completely eliminated by the combination of 5-HT_2_ and 5-HT_3_ antagonists (Fig. 5l), indicating that it was mediated by 5-HT_2_ and 5-HT_3_ receptors.

### 5-HT_2_ and 5-HT_3_ receptor agonists facilitate glutamate transmission in LHb neurons

We next investigated the effects of the 5-HT_2_ receptor agonist (10 μM mCPP, preferentially activates 5-HT_2C_ receptors), and the 5-HT_3_ receptor agonist (25 μM mCPBG), on sEPSCs. mCPP significantly increased the frequency and the amplitude of sEPSCs in all neurons tested in the LHbm (18/18, *p* < 0.001; Fig. 5A,C) and the majority of LHbl neurons (12/14, *t* = 4.8, *p* < 0.001; Fig. 5B,C). 5-HT_3_ receptor antagonist OND did not alter mCPP-induced increase of sEPSC frequency (LHbm: *n* = 8, *p* = 0.38; LHbl: *n* = 7, *p* = 0.33; Fig. 5D). Furthermore, mCPBG robustly potentiated sEPSCs in the majority of neurons in the LHbm (*n* = 30/32, frequency: *p* < 0.001; amplitude: *p* < 0.01; Fig. 5E,G) and LHbl (*n* = 13/15, frequency: *p* < 0.001; amplitude: *p* < 0.01; Fig. 5F,G). 5-HT_2_ antagonists (RIT plus SB200646) did not alter mCPBG-induced facilitation of sEPSC frequency (LHbm: *n* = 10, *p* = 0.34; LHbl: *n* = 9, *p* = 0.48; Fig. 5H). Notably, the potentiation induced by mCPP or mCPBG was similar in the LHbm and LHbl (all *p* > 0.5, unpaired *t*-test; Fig. 5C,G).

### 5-HT reuptake blocker increases glutamate transmission in LHb neurons

Given that LHb neurons are innervated by serotonergic fibers, we assessed whether endogenous 5-HT could facilitate sEPSCs in LHb neurons. A 10 min bath application of 10 μM citalopram, a highly selective 5-HT reuptake blocker, substantially increased sEPSC frequency in 10/11 LHbm neurons (*t* = 4.8, *p* < 0.001; Fig. 5I) and 10/16 LHbl neurons (*p* < 0.001; Fig. 5J). This effect was accompanied by an increased occurrence of larger sEPSCs (LHbm: by 5.2 ± 2.4%, *p* = 0.047; LHbl: by 4.3 ± 1.7%, *p* = 0.018; Fig. 5K). There was no significant difference in citalopram-induced augmentation on sEPSCs between the LHbm and LHbl (frequency: *t* = 1.24, *p* = 0.23; amplitude: *t* = 0.29, *p* = 0.77; Fig. 5K). In addition, citalopram-induced facilitation of sEPSC frequency in neurons in both the LHbm (*F*_3,33_ = 4.6, *p* = 0.009) and LHbl (*F*_3,40_ = 6.8, *p* < 0.001) was substantially attenuated by the 5-HT_2_ antagonists (RIT plus SB200646; LHbm: *p* = 0.038; LHbl: *p* = 0.032), and the 5-HT_3_ antagonist (OND; LHbm: *p* = 0.047; LHbl: *p* = 0.049), and almost completely abolished by the cocktail (RIT, SB200646 plus OND; LHbm: *p* = 0.003; LHbl: *p* < 0.001) (Fig. 5L).

## Discussion

We provide here the first electrophysiological evidence that 5-HT bi-directionally and differentially regulates glutamate transmission in the LHb. Furthermore, 5-HT’s facilitation of glutamate transmission is mediated by 5-HT_2_ and 5-HT_3_ receptors probably at the glutamatergic terminals. Finally, blockade of 5-HT reuptake facilitates glutamate transmission in most of the LHbm and LHbl neurons, suggesting that 5-HT may regulate glutamate transmission in the LHb under physiological conditions. Via altering glutamate transmission that regulates activity of LHb neurons, 5-HT may in turn alter the activity of raphe nuclei. Thus, 5-HT’s effects in the LHb may provide a feedback loop to the raphe nuclei, and may help maintain homeostatic balance of serotonergic function.

5-HT bi-directionally and differentially modulates glutamate transmission in the LHbm and LHbl neurons. Our result of 5-HT inhibition of glutamate transmission in LHbl neurons generally agreed with previous reports showing that 5-HT suppressed EPSCs in LHbl neurons in slices of rats^12^, and that 5-HT_1B_ agonist inhibited glutamate transmission in the LHb^28^. Our finding of 5-HT facilitation of glutamate transmission supports a recent *in vivo* study showing that intra-LHb injection of selective 5-HT_2C_ agonist Ro60-0175 increased the expression of depressive-like behaviors in rats, suggesting an excitatory effect of 5-HT in the LHb^27^. We extend this finding by demonstrating that 5-HT facilitated glutamate transmission in the majority of the LHbm neurons and a minority of the LHbl neurons.

A future study is needed to determine whether the different 5-HT effects are due to the difference in the neuronal phenotype. Using morphological analysis Weiss and Veh^31^ revealed four main categories of projection neurons randomly distributed throughout the LHb. However, electrophysiological characterization of neurons within the different categories demonstrated no significant differences between groups. Based on the pattern of spontaneous activity, neurons were classified as silent, tonic or bursting. The occurrence of distinctive firing modes was not related to topographic allocation. These investigators thus concluded that the formation of functional neuronal entities within the LHb may be achieved through defined synaptic inputs to particular neurons, rather than by individual neuronal morphologies and intrinsic membrane properties. By analyzing the gene expressions of neurotransmitter markers in the habenula, Aizawa, *et al.*^21^ showed that neurons in the LHb were almost uniformly glutamatergic. The current study revealed that EPSCs were recorded in almost all LHb neurons tested, and were sensitive to 5-HT. Thus, the different responses to 5-HT may be resulted from the heterogeneous expression of 5-HT receptors on the glutamatergic terminals in LHb neurons.

In the current study, we identified presynaptic 5-HT_2A/B/C_ and 5-HT_3_ receptors mediate 5-HT-induced facilitation of glutamate transmission. 5-HT potentiated the frequency and amplitude of sEPSCs. Tetrodotoxin completely abolished the effect on sEPSC amplitude, indicating that 5-HT’s effect on sEPSC amplitude depends on the action potentials. Action potential firing may increase the proportion of multiquantal events, thus skewing the amplitude distribution of synaptic currents towards larger size classes. 5-HT significantly increased the frequency but not the amplitude of mEPSCs, suggesting that 5-HT increases the probability of glutamate release, probably at the nerve terminals. In support, 5-HT increased the amplitude, and decreased the paired pulse ratio of the eEPSCs.

RNA labeling evidence indicates the existence of 5-HT_2_ receptors in the LHb^32^. Pharmacological evidence has confirmed the existence of 5-HT_1B_^28^ and 5-HT_2C_^27^ receptors in rat LHb. In both the LHbl and LHbm, we showed that 5-HT_2A/C_ antagonist ritanserin and 5-HT_2B/C_ antagonist SB200646 substantially attenuated 5-HT-induced facilitation of sEPSCs. Conversely, the 5-HT_2C_ agonist mCPP increased sEPSCs, in general agreement with a recent *in vivo* study^27^. Our data suggest that activation of 5-HT_2_ receptors increases glutamate release probability at the nerve terminals. The existence of 5-HT_3_ receptors was revealed by the application of ondansetron, which significantly attenuated 5-HT-induced facilitation of sEPSCs. Accordingly, bath application of the selective 5-HT_3_ agonist mCPBG increased sEPSCs. These data suggest that activation of 5-HT_3_ receptors increases glutamate release probability and partly mediates 5-HT’s facilitation of glutamate transmission in the LHb. Notably, the cocktail containing 5-HT_2_ and 5-HT_3_ receptor antagonists completely abolished 5-HT-induced potentiation of glutamate transmission. Our results thus revealed the presence of functional 5-HT_2A/B/C_ and 5-HT_3_ receptors that mediate 5-HT-induced facilitation of glutamate transmission in the LHb.

5-HT may modulate glutamate transmission in the LHb under physiological conditions. There is recent evidence^33^ that the 5-HT reuptake blocker citalopram affected the activity of the synapse connecting the basal ganglia with the LHb neuron. In general agreement with their finding, we found that citalopram potentiated glutamate transmission in LHb neurons, and the antagonists of 5-HT_2A/C,_ 5-HT_2B/C_, and 5-HT_3_ receptors significantly attenuated this potentiation, and the cocktail containing 5-HT_2_ and 5-HT_3_ receptor antagonists almost completely abolished this potentiation. These results suggest that citalopram, via the accumulated extracellular 5-HT, activates presynaptic 5-HT_2_ and 5-HT_3_ receptors, and increases probability of glutamate release.

Medial and lateral subdivisions of the LHb were recognized in an early rat study^34^. Ultrastructural^35^ and immunohistological^36^ studies in rats have defined as many as four medial and five lateral LHb regions. A corresponding subnuclear structure has been described in the mouse^37^. As mentioned, the neurons in the LHbl and LHbm are heterogeneous with different connectivity^31^. The subregions of the medial and lateral nuclei give rise to distinct projections to midbrain areas^15^, both in rat^14^,^30^ and mouse^38^. Since the LHbm sends glutamatergic projections mainly to interneurons in the raphe nucli^15^,^16^, LHbm activation may suppress raphe nuclei and 5-HT release. Conversely, since the LHbl mainly projects to the RMTg, which in turn sends GABAergic projections to the midbrain dopaminergic neurons and raphe serotoninergic neurons^13^,^14^, LHbl activation may reduce the activity of dopaminergic and serotoninergic neurons.

Notably, although 5-HT inhibited glutamate transmission in a subset of LHbl neurons, we have recently shown that 5-HT increases firing of the majority of the LHbl neurons by activating the postsynaptic 5-HT receptors^29^. We therefore speculate that the net effect of 5-HT may increase the activity of LHb neurons, which may in turn inhibit dopaminergic and serotoninergic neurons. This may provide an additional explanation at the cellular level for the previous functional discoveries; changes of the LHb activity lead to an opposite reaction of raphe cell activity, i.e., lesion of the LHb is followed by an increase in 5-HT in the dorsal raphe nucleus^20^,^39^,^40^,^41^; where electrical^42^,^43^ as well as chemical^16^,^25^ stimulation of the habenula markedly suppressed serotonergic neurons in the raphe nuclei.

The LHb itself is a hub in the forebrain, which plays critical roles in a variety of brain functions, such as depression, addiction, and sleep cycle disorders, as well as decision making^11^,^44^,^45^,^46^,^47^. At the neuronal level, integration of synaptic inputs and intrinsic properties sets the frequency and pattern of neuronal firing activity. Through the influence over glutamate transmission in the LHb, 5-HT may change the LHb output to the downstream regions. In view of the extensive innervation on midbrain monoaminergic nuclei by LHb neurons, these dual actions of 5-HT may have a profound effect on the operation of the entire midbrain network. Taken together, our data suggest a feedback loop between the LHb and the raphe nuclei, which may have a role in the balance of the reciprocal neural activity. Disruption of this fine natural balance may be involved in many neuropathology like drug abuse and mood disorders: depression and anxiety.

## Methods

### Animals

The Animal Care and Utilization Committee of Rutgers, the State University of New Jersey, in accordance with National Institutes of Health guidelines, approved all procedures, minimizing the number of animals used and their suffering. Sprague-Dawley (SD) rats (*n* = 200) at postnatal days 25–35 of both sexes were housed under standard conditions at 22–24 °C, 50–60% humidity, and a 12 h light/dark cycle. Food and water are available to all rats ad libidum unless otherwise indicated. Since the data from juvenile male rats did not differ significantly from those from female rats, the data were pooled.

### Brain slice preparation and electrophysiology

Coronal epithalamic slices (250 μm) were cut in ice-cold glycerol-based artificial cerebrospinal fluid (GaCSF) containing (in mM): 252 glycerol, 2.5 KCl, 1.25 NaH_2_PO_4_, 1 MgCl_2_, 2 CaCl_2_, 25 NaHCO_3_, 0.3 L-ascorbate, and 11 glucose, and saturated with 95%O_2_/5%CO_2_ (carbogen). Slices were incubated for >1-hr at 24–25 °C in carbogenated aCSF of similar composition as GaCSF, but with 126 mM NaCl replacing glycerol. Electrophysiological recordings (from ~700 LHb neurons) were performed at ~33 °C aCSF perfused at 1.5–2 ml/min, as described^48^. Patch pipettes (6–8 MΩ) were filled with internal solutions containing (in mM) 140 cesium methanesulfonate, 5 KCl, 2 MgCl_2_, 10 HEPES, 2 MgATP, 0.2 GTP for recordings under voltage-clamp. Both evoked and spontaneous events were recorded at a holding potential (V_H_) of −70 mV in the presence of gabazine (10 μM) and strychnine (1 μM), which block GABAA and glycine receptors, respectively. These events were blocked by 6,7-dinitroquinoxaline-2,3-dione (DNQX), an antagonist of α-amino-3-hydroxy-5-methylisoxazole-4-propionic acid (AMPA) receptors; indicating that they were excitatory postsynaptic currents (EPSCs), mediated by AMPA receptors. Electrical stimuli (100–200 μs in duration, 0.05 Hz) elicited EPSCs via a nichrome wire bipolar electrode positioned within 200 μm of the soma. Near the start of the recording an input/output curve was obtained and the stimulation was then set to 20–30% of the maximum, an intensity that resulted in stable responses with no failures. Paired eEPSCs were elicited with a pair of identical stimuli separated by an interval of 50 milliseconds.

### Drugs

We purchased common salts and 1-(3-Chlorophenyl) biguanide hydrochloride (mCPBG); gabazine; 1,2-bis(2-aminophenoxy) ethane-N,N,N,N-tetraacetic acid (BAPTA); SCH50911; 6,7-dinitroquinoxaline-2,3-dione (DNQX); tetrodotoxin (TTX); strychnine; SB200646 hydrochloride (SB200646); ritanserin (RIT); ondansetron hydrochloride dihydrate (OND); and 5-HT hydrochloride (5-HT) from Sigma-Aldrich Chemical Company (St Louis, MO, USA). Citalopram hydrobromide, WAY100635, and 1-(3-Chlorophenyl) piperazine hydrochloride (mCPP) from Tocris Bioscience (Ellisville, MO, USA).

### Data analysis and statistics

All data are expressed as means ± SEM. Baseline electrophysiological data were recorded for 10 min, before drug superfusion, and during the washout. To calculate the percent change in EPSC frequency/amplitude for a given cell, recordings during the initial control period (baseline) were averaged and normalized to 100%. Comparisons between the LHbm and LHbl were made using two-tailed unpaired Student’s *t*-tests. Possible significant differences in the percent distribution of EPSCs were compared by Chi-square test. The different concentrations of 5-HT on sEPSCs were analyzed using two-way ANOVA with “subregions” (LHbm vs LHbl) as between-group factors and “dose” (from 0.1 to 30 μM) as within subject factor. Tukey’s *post hoc* test was used for multiple dose comparisons. The effects of 5-HT antagonists on changes in sEPSCs induced by 5-HT/citalopram were assessed by paired *t*-test or one-way ANOVA. Dose-response data were fitted to the logistic equation: y = 100x^α^/(x^α^ + x_o_^α^), where y is the percentage change, x is the concentration of 5-HT, α the slope parameter, and x_o_ the 5-HT concentration which induces a half-maximal change. Values of *p* < 0.05 were considered significant.

## Additional Information

**How to cite this article**: Xie, G. *et al.* Serotonin modulates glutamatergic transmission to neurons in the lateral habenula. *Sci. Rep.* **6**, 23798; doi: 10.1038/srep23798 (2016).

## Figures and Tables

**Table 1:** 5-HT (10 μM) induced changes in glutamatergic transmission in the LHbm and LHbl neurons.

		Cases Distribution		*p*-Value (LHbm vs LHbl)	% Changes		*p*-Value (LHbm vs LHbl)
EPSC		LHbm	LHbl		LHbm	LHbl	
sEPSC frequency	↓	13/67	37/70	*p* < 0.001	40.6 ± 6.9^###^	45.8 ± 4.7^###^	ns
	↑	50/67	27/70		153.4 ± 14.6^###^	108.2 ± 13.5^##^	*p* = 0.029
	—	4/67	6/70		—	—	—
sEPSC amplitude	↓	13/67	37/70	*p* < 0.001	29.5 ± 6.4^###^	34.4 ± 5.2^###^	ns
	↑	50/67	27/70		13.4 ± 1.9^#^	12.5 ± 2.3	ns
eEPSC amplitude	↓	3/21	12/24	*p* = 0.025	42.7 ± 8.6^##^	41.9 ± 5.6^###^	ns
	↑	17/21	10/24		56.7 ± 8.5^###^	32.3 ± 4.6^###^	*p* = 0.048
	—	1/21	2/24		—	—	—

Values are expressed as mean ± SEM., ^#^*p* < 0.05, ^##^*p* < 0.01, ^###^*p* < 0.001 vs baseline; ↓decrease; ↑increase; ─ no change. ns means no significant difference.
